# Impaired detection of omicron by SARS-CoV-2 rapid antigen tests

**DOI:** 10.1007/s00430-022-00730-z

**Published:** 2022-02-20

**Authors:** Andreas Osterman, Irina Badell, Elif Basara, Marcel Stern, Fabian Kriesel, Marwa Eletreby, Gamze Naz Öztan, Melanie Huber, Hanna Autenrieth, Ricarda Knabe, Patricia M. Späth, Maximilian Muenchhoff, Alexander Graf, Stefan Krebs, Helmut Blum, Jürgen Durner, Ludwig Czibere, Christopher Dächert, Lars Kaderali, Hanna-Mari Baldauf, Oliver T. Keppler

**Affiliations:** 1 Max Von Pettenkofer Institute and Gene Center, Virology, National Reference Center for Retroviruses, LMU München, Feodor-Lynen-Str. 23, 81377 Munich, Germany; 2 German Center for Infection Research (DZIF), Partner Site, Munich, Germany; 3 COVID-19 Registry of the LMU Munich (CORKUM), University Hospital, LMU Munich, Munich, Germany; 4 Laboratory for Functional Genome Analysis, Gene Center, LMU München, Munich, Germany; 5 Labor Becker MVZ GbR, Munich, Germany; 6 Department of Conservative Dentistry and Periodontology, University Hospital, LMU München, Goethestr. 70, 80336 Munich, Germany; 7 Institute of Bioinformatics, University Medicine Greifswald, Felix-Hausdorff-Str. 8, 17475 Greifswald, Germany

**Keywords:** SARS-CoV-2 rapid antigen test, Nucleocapsid protein, Diagnostic test, Sensitivity, Specificity, VoC, Lateral flow

## Abstract

Since autumn 2020, rapid antigen tests (RATs) have been implemented in several countries as an important pillar of the national testing strategy to rapidly screen for infections on site during the SARS-CoV-2 pandemic. The current surge in infection rates around the globe is driven by the variant of concern (VoC) omicron (B.1.1.529). Here, we evaluated the performance of nine SARS-CoV-2 RATs in a single-centre laboratory study. We examined a total of 115 SARS-CoV-2 PCR-negative and 166 SARS-CoV-2 PCR-positive respiratory swab samples (101 omicron, 65 delta (B.1.617.2)) collected from October 2021 until January 2022 as well as cell culture-expanded clinical isolates of both VoCs. In an assessment of the analytical sensitivity in clinical specimen, the 50% limit of detection (LoD50) ranged from 1.77 × 10^6^ to 7.03 × 10^7^ RNA copies subjected to the RAT for omicron compared to 1.32 × 10^5^ to 2.05 × 10^6^ for delta. To score positive in these point-of-care tests, up to 10-fold (LoD50) or 101-fold (LoD95) higher virus loads were required for omicron- compared to delta-containing samples. The rates of true positive test results for omicron samples in the highest virus load category (Ct values < 25) ranged between 31.4 and 77.8%, while they dropped to 0–8.3% for samples with intermediate Ct values (25–30). Of note, testing of expanded virus stocks suggested a comparable RAT sensitivity of both VoCs, questioning the predictive value of this type of in vitro-studies for clinical performance. Given their importance for national test strategies in the current omicron wave, awareness must be increased for the reduced detection rate of omicron infections by RATs and a short list of suitable RATs that fulfill the minimal requirements of performance should be rapidly disclosed.

**Supplementary Information:**

The online version contains supplementary material available at 10.1007/s00430-022-00730-z.

## Introduction

During the SARS-CoV-2 pandemic, new variants of the virus continue to emerge. The variant of concern (VoC) omicron was first detected in November 2021 in Southern Africa. Omicron’s ability for immune escape [1–5] and high transmissibility in the population [6–10] have resulted in omicron’s rapid spread around the globe [11, 12] and have fueled the pandemic with numbers of new infections reaching all-time highs despite increasing COVID-19 vaccination rates. In early February 2022, numbers of mostly unvaccinated, at-risk COVID-19 patients with omicron that require hospitalization or even intensive care treatment are high in some countries, including Israel and the United States. Omicron is characterized by a large number of mutations in the spike protein [13] and currently has two prominent sub-lineages based on Pangolin nomenclature, namely BA.1 and BA.2.

In many countries, rapid antigen tests (RATs) for the detection of SARS-CoV-2 continue to be a central component of national testing strategies offering quick, inexpensive and laboratory-independent, point-of-care diagnostics. However, the evaluation by independent laboratories [14–25] and a Cochrane meta-analysis [26] have indicated a highly variable performance of RATs resulting in an ongoing controversy over these tests’ utility for the detection of acute SARS-CoV-2-infected individuals in different settings relevant for clinical diagnosis and containment strategies.

Some of these RATs have been approved for use by layperson and are frequently used as a tool for public health interventions. In light of the somewhat limited capacities of laboratory-based RT-qPCR for the quantitative detection of SARS-CoV-2 RNA, test results from RATs alone are currently being suggested by national health authorities both to reliably diagnose COVID-19 or to document an “uninfected” or “non-infectious” status for ending quarantine or isolation of persons without symptoms, respectively.

The nucleocapsid protein is most commonly detected by SARS-CoV-2 antigen tests [13]. Since the majority of RATs was developed prior to the emergence of VoCs, the latter carrying different mutational patterns in the nucleocapsid protein [19] (Suppl. Table 1), it is of utmost importance to perform VoC-specific RAT evaluations. In omicron, besides more than 30 non-synonymous mutations in the spike protein, the nucleocapsid protein of the BA.1 sub-lineage carries four mutations compared to the reference sequence of the Wuhan-hu-1 virus, i.e. P13L, DEL31/33, R203K and G204R, and the additional mutation S413R is present in BA.2 (Suppl. Table 1). Three of these mutations (P13L, DEL31/33 and S413R) are unique to VoC omicron compared to alpha, beta, gamma or delta, rendering predictions of the performance of RATs difficult.

Notably, first reports on the clinical and analytical performance of RATs for omicron are partly contradictory: a high-risk occupational case cohort of 30 individuals with daily testing during an omicron outbreak in December 2021 showed a median of two days of apparently false-negative RAT results with four recorded transmission events during the period preceding the positive PCR result [27]. A preprint study reports on 296 persons seeking COVID-19 testing at a walk-up San Francisco community site in January 2022, who tested positive by RT-qPCR. In these individuals, simultaneous nasal rapid antigen testing with BinaxNOW™ detected 95.2% of high viral load-omicron cases and 65.2% of all PCR-positive cases [28]. A comparative study on isolates classified as delta or omicron, that had been expanded in tissue culture, found no VoC-specific differences in the analytical sensitivity of ten RATs, concluding the effectiveness of these antigen lateral flow tests for omicron [29]. Other investigators have suggested that an analytical and comparative validation with cultured VoCs might be a proxy for clinical accuracy, but also stated that this cannot replace clinical evaluations [14].

The aim of the current study was to compare nine different SARS-CoV-2 RATs that all detect the nucleocapsid protein of SARS-CoV-2, for their analytical performance using both clinical respiratory material and cultured viruses. We evaluated respiratory specimen, identified as either delta or omicron that had been collected from patients seen at different hospitals, nursing homes, outpatient clinics, COVID-19 testing centers or seen by primary care physicians in the Greater Munich area during the fourth and fifth pandemic wave in Germany in the period October 2021 until January 2022. In addition, dilution series of isolates of delta and omicron that had been expanded in cell culture in a biosafety level 3 laboratory, were assessed.

## Materials and methods

### Respiratory swabs

In the period October 30, 2021 to January 17, 2022 (delta) and November 26, 2021 to January 19, 2022 (omicron), health care professionals collected respiratory swabs (nasopharyngeal or nasal) from individuals who were seen on clinical units or in the emergency room of the LMU Klinikum, the second-largest University Hospital in Germany, and three teaching hospitals of the LMU Munich (Helios Amper Hospital Dachau, Helios Hospital München West and Helios Hospital München Perlach). Furthermore, SARS-CoV-2 in respiratory swabs identified as omicron by Labor Becker MVZ GbR in Munich, Germany, a diagnostic laboratory that receives samples from regional hospitals, COVID-19 testing centers, nursing homes and primary care physicians' offices, completed the study panel. All samples from this period were randomly included in the study depending on the availability of sufficient sample volume. No information on the persons’ vaccination status, previous infections, symptoms or clinical course were available. For this study, flocked swabs were collected in different liquid transport media and analyzed by RT-qPCR for SARS-CoV-2 RNA. Dry swabs were resuspended in sterile 0.9% NaCl. Patient samples in liquid transport medium with denaturing potential were excluded from the study. All samples with a Cp/Ct value < 40 by RT-qPCR, which was quantified less than 24 h after sample collection and under accredited diagnostic laboratory conditions, were scored “SARS-CoV-2-positive”. Original respiratory swabs and transport media were stored at 2–8 °C for up to 1 week. If longer storage periods were required, samples were frozen at − 20 °C and thawed once before antigen testing was performed. 166 SARS-CoV-2-PCR-positive and 115 PCR-negative respiratory samples were analyzed in this study.

### SARS-CoV-2 antigen tests

**Fig. 1:**
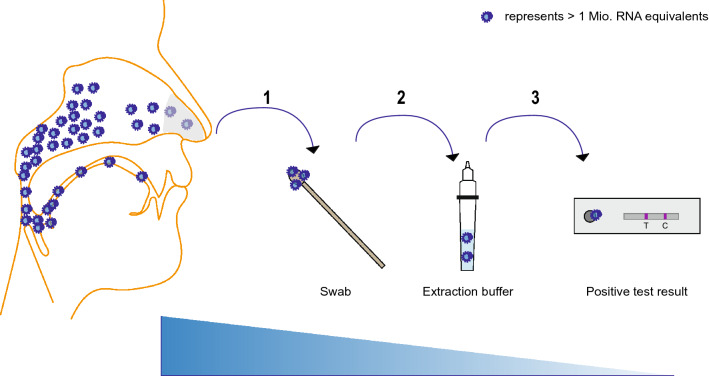
Schematic overview of the nasal swab sampling and testing procedure. Step 1: viral particles are collected with the provided collecting device (swab) from the anterior part of the nasal cavity. In general, the test sensitivity is influenced by the amount of nucleocapsid antigen collected with the swab from nasal mucosa and secretions. Step 2: antigen-containing sample material is eluted from the swab into an extraction buffer. The sensitivity of the assay can be affected by the efficiency of the physical elution of the antigen from the collection device. Step 3: extracted nucleocapsid antigen is applied onto the rapid antigen test cassette. In this step, manufacturer-specific recommendations for the volume of antigen-containing buffer that are applied to the test cassette (known as “input ratio”) exist. The amount of “RNA copies subjected to assay” investigated in this study refers to an input equivalent at step 1

The methodological procedure for the evaluation of the RATs in this study follows the publication on the comparative sensitivity evaluation for CE-marked rapid diagnostic tests for SARS-CoV-2 antigen as "corresponding to the current state-of-the-art" of the Paul-Ehrlich-Institute [21]. For testing a particular specimen, a 50 µl aliquot was added and completely absorbed using the sampling devices (swab) supplied with the respective kit. This corresponds to a spike-in after step 1 in Fig. 1. Swabs were then eluted in the test-specific buffer, following the respective manufacturer's instructions. After application of the specimen/buffer solution to the test cassette, visual reading of the control and target lines was performed after a 15 min-incubation period. The appearance of the RDT control line was mandatory for a valid test result. Invalid tests were documented and repeated if possible. Any visible colored band in the test area was considered a positive result regardless of the intensity of the band. Scoring the RAT was performed under constant lighting conditions and by a trained person who was blinded to the preceding RT-qPCR result of the sample. This procedure deviates in part from manufacturer specifications regarding the use of liquid transport medium rather than direct swabs and the fact that the sample was not immediately inserted into the assay. These study design-related adaptations in the protocol are unlikely to affect the comparative assessment of the RATs for VoCs delta and omicron. For comparability, this study reports the number of SARS-CoV-2 RNA copies subjected to each test (determined by absolute quantification of RT-qPCR—see below).

Eight of the RATs examined in this study are recommended by the manufacturers for self-testing and were listed as a CE-labeled antigen test by the Bundesinstitut für Arzneimittel und Medizinprodukte (BfArM) in Germany at the time of purchase, fulfilling the minimum criteria required for these test devices in Germany according to the information provided by the manufacturers. Of note, these eight assays investigated were among the first rapid SARS-CoV-2 antigen tests approved by BfarM for lay use in March 2021.

In detail, the products and manufacturers are: for test 1: FUJIFILM COVID-19 Ag Test (Fujifilm Cooperation), research-use-only kits were provided for this study by the manufacturer. Except for the labels, these are identical to the final product, which meets the above criteria and was introduced after approval. Test 2: Novel Coronavirus 2019-nCoV Antigen Test (Colloidal gold) (Beijing Hotgen Biotech Co., Ltd.); test 3: NanoRepro SARS-CoV-2 Antigen Schnelltest (Viromed) (NanoRepro AG); test 4: CLINITEST Rapid COVID-19 Antigen Test (Healgen Scientific LLC); test 5: Lyher Novel Coronavirus (COVID-19) Antigen Test Kit (Colloidal Gold) (Hangzhou Laihe Biotech Co., Ltd.); test 6: COVID-19 Ag BSS self-test (Biosynex Swiss SA); test 7: rapid SARS-CoV-2 Antigen Test Card (Xiamen Boson Biotech Co., Ltd.); test 8: rapid SARS-CoV-2 Antigen Test Card (MP Biomedicals Germany GmbH) and test 9: Medicovid-AG SARS-CoV-2 Antigen Rapid Test Card-nasal (Xiamen Boson Biotech Co., Ltd.). Tests 7 and 9 appear to be the same antigen cassette product from one manufacturer (Ref No.: 1N40C5 and 1N40C5-4), but they may differ in other kit components (swab and buffer) in addition to package size. Eight of these nine RATs have been evaluated by the Paul-Ehrlich-Institute [21] and the European Commission [30]. An overview of the RATs used in the current study and their characteristics is provided in Suppl. Table 2.

### Quantitative viral load determination

In the accredited routine diagnostics laboratory of the Max von Pettenkofer Institute, the following RT-qPCR assays were used and quantified as described previously [19] using the respective formulas: the nucleocapsid (N1) reaction (Center for Disease Control (CDC) protocol [31] ($x={\mathrm{e}}^{\frac{(y-48.597)}{-1.461}}$) on a LightCycler 480 system, the Roche Cobas SARS-CoV-2 E-Gen reaction on a Cobas 6800 system ($x={\mathrm{e}}^{\frac{(y-44.576)}{-1.401}}$) or the Xpert Xpress SARS-CoV-2 ($x={\mathrm{e}}^{\frac{(y-50.859)}{-1.887}}$), Xpert Xpress SARS-CoV-2/Flu/RSV ($x={\mathrm{e}}^{\frac{(y-45.904)}{-1.5}}$) and Xpert Xpress CoV-2/Flu/RSV*plus* ($x={\mathrm{e}}^{\frac{(y-46.747)}{-1.501}}$) run on a GeneXpert System, with x equals Geq per ml and y equals the Ct/Cq value. For nucleic acid extraction, the QIAsymphony DSP Virus/Pathogen Kit was used with the QIAsymphony instrument from QIAGEN GmbH. In general, the calculations for quantification do not take into account variability between separate RT-qPCR runs. However, since this variability applies to all study groups, they do not affect the interpretation of the results in this study.

### SARS-CoV-2 variant-specific PCR and whole-genome sequencing

Protocols for SARS-CoV-2 variant-specific PCR have been reported [32]. In brief, nucleic acids from patients’ respiratory samples were extracted and eluates used for melting curve analyses using commercially available PCR kits from two vendors. For whole genome sequencing, amplicon pools covering the SARS-CoV-2 genome were prepared according to the ARTIC network nCoV-2019 sequencing protocol v2 and analyzed utilizing the Artic bioinformatics protocol [33]. The consensus sequences and associated sample metadata were uploaded to the GISAID repository.

### Expansion of SARS-CoV-2 from patient material

SARS-CoV-2 was expanded as reported [5]. In brief, for generation of high titer SARS-CoV-2 virus stocks, Vero-E6 cells were challenged with respiratory specimens containing either delta or omicron. Virus stocks were harvested three days post infection when approximately 50% of cells were detached displaying a strong cytopathic effect. Supernatants were cleared and stored at −80 °C. Expanded stocks were analyzed by whole genome sequencing (B.1.617.2: GISAID 3233464; B.1.1.529: GISAID 7808190) and RNA copies per mL were determined as the mean from three independent biological experiments with technical unicates or duplicates.

### Statistical analyses

Statistical analysis was performed in R version 4.1.2. Binomial confidence intervals for sensitivities and specificities were computed using the Wilson score interval. To further analyze analytical sensitivities, we used logistic regression, with viral loads and RNA copy numbers subjected to the test as independent and test outcomes as the dependent variable, yielding detection probabilities for each viral load level.

## Results

### Evaluation of RAT specificity

To evaluate the specificity of all RATs in a comparable experimental setting (Fig. 1), test collection devices (swabs) were individually incubated with 50 µl each of the individual 115 PCR-negative nasal/nasopharyngeal swabs and applied to the individual assay’s extraction buffer. Under these conditions, the specificity of all nine RATs was 100% (CI 96.3–100%) (Suppl. Table 3).

### Characterization of respiratory specimens from COVID-19 patients containing VoCs delta or omicron

**Fig. 2:**
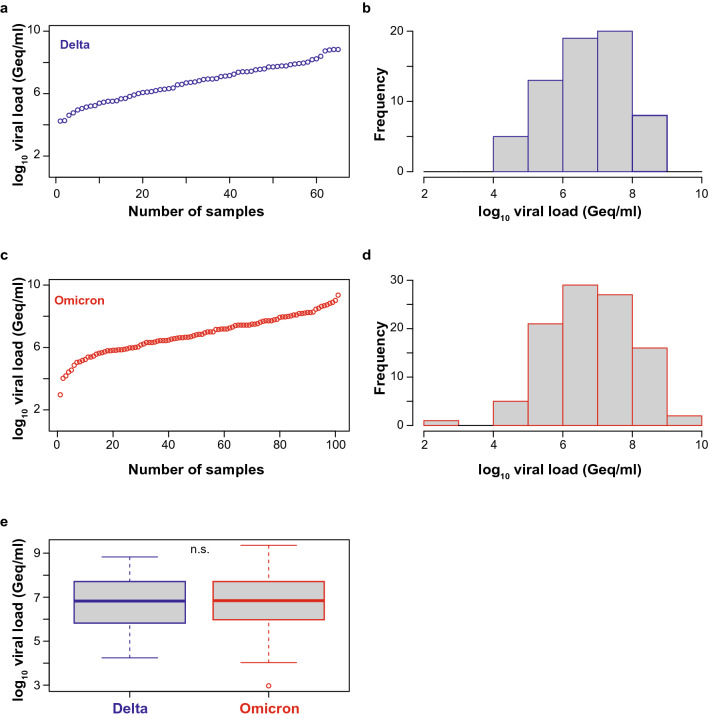
SARS-CoV-2 viral load distribution of respiratory samples included in the study containing either delta or omicron. **a**, **c** Shown is the log10 viral load (Geq/ml) distribution of all 65 delta (**a**) and all 101 omicron (**c**) sorted by ascending magnitude from left to right. Each dot indicates one patient and the sample ID is indicated. **b**,** d** Histogram of the viral load distribution in specimen containing delta (**b**) or omicron (**d**) categorized into the indicated ranges of log10 viral load. Each bar depicts the number of samples in the respective viral load range. **e** The horizontal line in the box plots shows the median of the samples shown in **a** and **c**, bound between upper and lower quartiles, and whiskers between minimum and maximum are indicated. *n.s*. = not significant by Wilcoxon rank sum test with continuity correction and by two-sample Kolmogorov–Smirnov test

Next, we quantified the viral load in PCR-positive nasopharyngeal swabs, of which 65 were classified as delta (B.1.617.2) and 101 as omicron (B.1.1.529), respectively, by variant-specific PCR and next-generation sequencing [32]. The omicron sequences in all respiratory swabs were classified as belonging to the BA.1 sublineage. The viral loads of delta specimen ranged from 1.74 × 10^4^ to 6.72 × 10^8^ Geq/ml (median 6.67 × 10^6^ Geq/ml, interquartile range 6.61 × 10^5^ – 5.14 × 10^7^ Geq/ml, median absolute deviation 9.73 × 10^6^ Geq/ml) (Fig. 2a, b, e), the viral loads of omicron specimen ranged from 9.3 × 10^2^ to 2.26 × 10^9^ Geq/ml (median 6.97 × 10^6^ Geq/ml, interquartile range 9.45 × 10^5^–5.14 × 10^7^ Geq/ml, median absolute deviation 1.02 × 10^7^ Geq/ml) (Fig. 2c, d, e). Importantly, the median and distribution of viral loads were comparable and statistically indistinguishable between both groups of VoC-containing respiratory samples (Fig. 2e), allowing a direct comparison of the analytical sensitivity of RATs for the detection of these two most recently emerged VoCs.

### Comparative analysis of analytical RAT sensitivity for VoCs delta and omicron in clinical specimens

We used this panel of SARS-CoV-2 RT-qPCR-positive respiratory specimen to determine the analytical sensitivity of the nine RATs for the detection of delta and omicron in a direct comparison. The overall sensitivities for delta-containing specimen ranged between 34.92 and 58.46% (Table 1), corresponding to 22 of 63 and 38 of 65 samples, respectively, that were tested true positive. For omicron-containing specimens, the overall sensitivities ranged from 22.22 to 57.43% (Table 2), corresponding to 22 of 99 and 58 of 101 SARS-CoV-2 PCR-positive samples, respectively, that were correctly scored.

**Table 1:** Determination of assay sensitivity for nine SARS-CoV-2 rapid antigen tests in SARS-CoV-2 PCR-positive respiratory swabs classified as delta

Assay	Sensitivity (%)	95% CI (%)	True positive/total
Test 1	34.92	24.33–47.25	22/63
Test 2	56.25	44.09–67.71	36/64
Test 3	53.85	41.85–65.41	35/65
Test 4	55.38	43.34–66.83	36/65
Test 5	58.46	46.34–69.64	38/65
Test 6	55.38	43.34–66.83	36/65
Test 7	49.21	37.27–61.24	31/63
Test 8	58.46	46.34–69.64	38/65
Test 9	55.38	43.34–66.83	36/65

Binomial confidence intervals were computed using the Wilson score interval

**Table 2:** Determination of assay sensitivity for nine SARS-CoV-2 rapid antigen tests in SARS-CoV-2 PCR-positive respiratory swabs classified as omicron

Assay	Sensitivity (%)	95% CI (%)	True positive/total
Test 1	22.22	15.16–31.36	22/99
Test 2	35.64	26.99–45.35	36/101
Test 3	31.68	23.42–41.29	32/101
Test 4	35.64	26.99–45.35	36/101
Test 5	47.52	38.06–57.18	48/101
Test 6	43.00	33.73–52.78	43/100
Test 7	29.70	21.67–39.23	30/101
Test 8	51.49	41.86–61.00	52/101
Test 9	57.43	47.69–66.62	58/101

Binomial confidence intervals were computed using the Wilson score interval

**Fig. 3:**
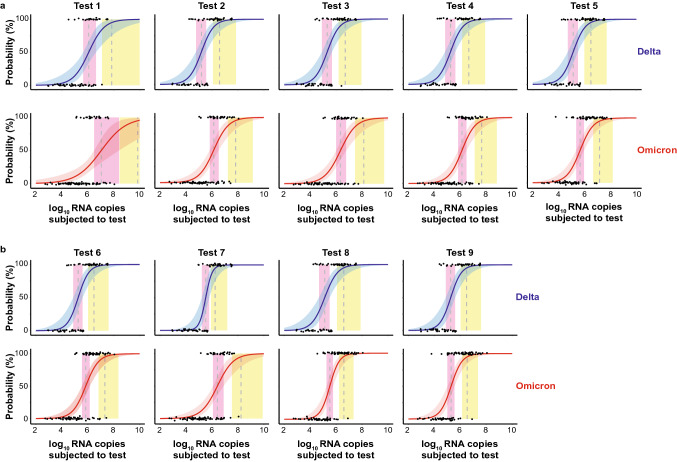
Limit of detection analyses of respiratory samples positive for either delta (top panels) or omicron (bottom panels) by RT-qPCR for nine SARS-CoV-2 RATs. **a** test #1–5, **b** test #6–9. The log10 RNA copies subjected to the test of quantified samples on the *x* axis were plotted against a positive (+ 1) or negative (0) test outcome on the *y* axis. For readability of the figure, slight normal jitter was added to the *y* values. Blue (delta) and red (omicron) curves, respectively, show logistic regressions of the viral load on the test outcome; vertical dashed lines indicate log10 RNA copies subjected to the test at which 50% (LoD50) and 95% (LoD95), respectively, of the samples are expected positive based on the regression results. Significant differences for LoD50: test 2, 3, 4 and 7. Significant difference for LoD95: test 7

We determined the 50% (dotted line in pink vertical area) and 95% (dotted line in yellow vertical area) limits of detection (LoD) based on a logistic regression model as recently reported [16] (Fig. 3a, b). For test 1, the RNA copy numbers for LoD50 and LoD95 were 1.19 × 10^6^ and 7.03 × 10^7^ for delta-containing specimen, respectively. The detection of omicron-containing samples was in contrast impaired: the LoD50 and LoD95 values corresponded to 1.11 × 10^7^ and 7.12 × 10^9^ RNA copies, respectively, thus requiring 9- to 101-fold higher RNA copy numbers than those for delta-containing specimens to reach these detection thresholds. For test 2, the LoD50 and LoD95 values for delta-containing samples corresponded to 1.54 × 10^5^ and 3.86 × 10^6^ RNA copy numbers, respectively. Those for omicron-containing specimens were 9- to 17-fold higher with 1.38 × 10^6^ and 6.64 × 10^7^ RNA copy numbers, respectively. The performance of test 3 was comparable to test 2 with LoD50 and LoD95 values for delta-containing samples of 2.05 × 10^5^ and 5.04 × 10^6^ RNA copy numbers, respectively, and for omicron-containing samples of 2.12 × 10^6^ and 1.35 × 10^8^ RNA copy numbers, respectively, i.e. ranging 10- to 27-fold higher for the latter. The SARS-CoV-2 RNA copy numbers corresponding to LoD50 and LoD95 for test 4 for delta- versus omicron-containing specimens were 8- to tenfold higher for the latter with 1.77 × 10^5^ and 4.52 × 10^6^ versus 1.33 × 10^6^ and 4.37 × 10^7^, respectively. For test 5, the LoD50 and LoD95 values for delta-containing samples corresponded to 1.34 × 10^5^ and 3.08 × 10^6^ RNA copies, respectively. These LoD values were 3- and 5-times higher for omicron-containing samples with 4.54 × 10^5^ and 1.43 × 10^7^ RNA copies, respectively. The LoD50 and LoD95 values for test 6 were 1.82 × 10^5^ and 3.02 × 10^6^ for delta- and 6.93 × 10^5^ and 2.08 × 10^7^ for omicron-containing specimen, respectively, corresponding to a 4- to sevenfold difference. For test 7, the RNA copies with 50% and 95% detection rates determined for delta-containing specimens were 3.31 × 10^5^ and 1.77 × 10^6^, respectively. Detection of omicron was similar to test 1 and strongly impaired as LoD values were 8- to 99-fold higher with 2.62 × 10^6^ and 1.75 × 10^8^ RNA copies, respectively. The relative performance of test 8 was among the best of the RATs tested in this study: the LoD50 and LoD95 values for delta and omicron differed at maximum by a factor of 2: values for delta-containing samples corresponded to 1.32 × 10^5^ (LoD50) and 3.86 × 10^6^ (LoD95), and for omicron samples 3.24 × 10^5^ and 3.94 × 10^6^ RNA copies, respectively. Test 9 performed similarly well in our analysis: the LoD50 and LoD95 for delta samples were 1.82 × 10^5^ and 3.02 × 10^6^ RNA copies and for omicron samples 1.94 × 10^5^ and 3.41 × 10^6^, respectively.

In summary, a reduction in the analytical sensitivity for the detection of omicron- compared to delta-containing respiratory specimens, determined by the LoD50 and LoD95, was seen for eight of the nine RATs with factors of up to 10.3-fold and up to 101-fold more virus genome equivalents, respectively, required to score positive, reaching statistical significance for tests 2, 3, 4 and 7. This suggests a general impairment for a virus load-stratified detection of an omicron infection compared to a delta infection by SARS-CoV-2 antigen testing with a RAT-specific degree of sensitivity reduction.

### Analytical RAT sensitivity in cell culture-expanded virus stocks

Based on an initial report of comparable sensitivity for in vitro-expanded virus stocks [29], we assessed in parallel cell-culture expanded delta and omicron stocks. The expanded delta isolate (GISAID 3233464) carries the D63G, R203M and D377Y mutations in the nucleocapsid protein as well as the commonly reported G215C mutation [34]. The expanded omicron isolate (GISAID 7808190; BA.1 sublineage) carries the expected P13L, del31/33, R203K and G204R mutations. Neither culture medium alone nor the virus-inactivating detergent Triton X-100 had an impact on the performance of the RATs (not shown).

As shown in Suppl. Figure 1, rapid antigen tests 1, 5, 8 and 9 were able to detect delta and omicron virus stocks down to 2.5 × 10^6^ RNA copies, whereas the other five tests were less sensitive requiring up to 8-times more SARS-CoV-2 RNA to score positive. Interestingly, there was a trend for a slightly better detection of cell-culture expanded omicron compared to delta, in particular for tests 2, 4, 6 and 7. In summary, this analysis confirms first studies by other laboratories [14, 35] indicating a comparable detection of in vitro-expanded omicron and delta virus stocks by rapid antigen testing. However, this stands in clear contrast to the above evaluation of clinical respiratory specimens, which show an impaired sensitivity for the detection of an omicron infection in COVID-19 patients with comparable viral loads in respiratory specimens.

### Comparative, Ct value-stratified evaluation of analytical RAT sensitivity

Next, we sought to directly compare the analytical sensitivities of the nine RATs for the detection of “non-delta and non-omicron” SARS-CoV-2, evaluated and published by the Paul-Ehrlich-Institute, to VoCs delta or omicron assessed in our study in respiratory specimen in predefined corridors of SARS-CoV-2 RNA copy numbers. To this end, we harmonized the corresponding Ct/Cp values between our study results and those reported by Puyskens et al*.* and Scheiblauer et al*.* [21, 36]. This allowed us to plot and compare the percentages of test samples that scored positive in one of the three categories of Ct/Cp values, i.e. < 25, 25–30 and > 30.

The following observations were made (Table 3): (1) for the seven RATs, for which all three variant data sets were available, the overall sensitivity of the “non-delta and non-omicron” SARS-CoV-2 scored highest in four RATs, closely followed by three RATs for delta. (2) In eight out of nine RATs, the overall sensitivity was higher for delta- compared to omicron-containing respiratory samples. (3) In the highest viral load category with Ct values < 25, omicron detected the lowest percentage of cases in eight out of nine RATs with test 9 posing the exception. (4) In the intermediate viral load corridor reflected by Ct values 25–30, none of the respiratory specimen scored positive in six of the RATs, and with low percentages of 4.2–8.3% for tests 5, 8 and 9. (5) Samples with Ct values > 30 were generally not detected with a single exception for the “non-delta and non-omicron” specimen group (Table 3). In summary, these results indicate that the analytical sensitivity of RATs for omicron-containing respiratory samples cannot be deduced from previous evaluations of other SARS-CoV-2 variants. For the majority of RATs evaluated in this study, an impaired detection of the emergent VoC omicron compared to previous variants, including delta, was noted.

**Table 3:** Comparative evaluation of the analytical sensitivity of nine SARS-CoV-2 rapid antigen tests stratified for Ct/Cp value ranges based on studies by the Paul-Ehrlich-Institute (non-delta/non-omicron*) and the current study for respiratory samples containing VoCs delta and omicron

	*N*	Ct < 25 (%)^1^	Ct 25–30 (%)^1^	Ct > 30 (%)^1^	Overall sensitivity (%)
*Test 1*					
Non-Delta/non-Omicron*	n.a	85.0	10.0	0	38.0
Delta	63	50.0	5.6	0	34.9
Omicron	99	31.4	0	0	22.2
*Test 2*					
Non-Delta/non-Omicron*	n.a	100	47.8	0	56.0
Delta	64	74.4	22.2	0	56.2
Omicron	101	50.0	0	0	35.6
*Test 3*					
Non-Delta/non-Omicron*	n.a	94.1	–	–	–
Delta	65	72.7	16.7	0	53.9
Omicron	101	44.4	0	0	31.7
*Test 4*					
Non-Delta/non-Omicron*	n.a	100	87	0	76.0
Delta	65	72.7	22.2	0	55.4
Omicron	101	50.0	0	0	35.6
*Test 5*					
Non-Delta/non-Omicron*	n.a	94.4	17.4	0	42.0
Delta	65	77.3	22.2	0	58.5
Omicron	101	63.9	8.3	0	47.5
*Test 6*					
Non-Delta/non-Omicron*	n.a	100	78.3	11.1	74.0
Delta	65	75.0	16.7	0	55.4
Omicron	100	60.6	0	0	43.0
*Test 7*					
Non-Delta/non-Omicron*	n.a	100	43.5	0	54.0
Delta	63	72.1	0	0	49.2
Omicron	101	41.7	0	0	29.7
*Test 8*					
Non-Delta/non-Omicron*	n.a	100	43.5	0	54.0
Delta	65	75.0	27.8	0	58.5
Omicron	101	70.8	4.2	0	51.5
*Test 9*					
Non-Delta/non-Omicron*	n.a	–	–	–	–
Delta	65	75.0	16.7	0	55.4
Omicron	101	77.8	8.3	0	57.4

^*^Data published by Puyskens et al*.* (collected between March and September 2020 (Panel 1V1; [36]) and Scheiblauer et al*.* (additionally collected between October 2020 and January 2021 (Panel 1V2); [21]) and “SARS-CoV-2 antigen rapid diagnostic tests passing the sensitivity criteria” of the Paul-Ehrlich-Institute (date of access: 31.1.22)

^1^The Ct values of test specimens in the study performed by the Paul-Ehrlich-Institute and our study were harmonized to allow a direct comparison of data sets. Harmonization was achieved based on the publication by Scheiblauer et al*.* and the formula derived to convert virus loads into Ct values:

y = − 1.455lnx + 41.154 with x equals Geq per ml and y equals the Ct/Cq value. *n.a*. not available;– these test results were no longer included in the latest data set released by the Paul-Ehrlich-Institute on January 31, 2022

## Discussion

In the current study, we evaluated the performance of nine commercially available SARS-CoV-2 raid antigen tests that were launched on the European market during the coronavirus pandemic for the detection of the viral nucleocapsid protein. We find that for the majority of RATs evaluated, detection of omicron is impaired compared to delta, requiring up to 10.3-fold and up to 101-fold more SARS-CoV-2 RNA of omicron in respiratory specimens to score positive in LoD50 or LoD95 analyses, respectively.

We examined two groups of specimens: the first group encompassed respiratory samples collected from patients in the period October 2021 until January 2022. A total of 115 SARS-CoV-2 PCR-negative and 166 SARS-CoV-2 PCR-positive specimen (65 delta, 101 omicron) were tested. The second group consisted of cell culture-expanded clinical isolates of these VoCs. The latter was important to assess the predictive value of sensitivity evaluations of RATs using SARS-CoV-2 variants expanded in tissue culture as a potentially rapid proxy for their diagnostic performance.

Limitations of the current study include the lack of information on the vaccination status, previous infections, symptoms or stage of COVID-19 of individuals from whom the respiratory swabs were taken. We focused on the analytical sensitivity in relation to the viral loads in respiratory swabs to allow a direct comparison of specimens containing omicron and delta (the current study) and “non-omicron/non-delta” SARS-CoV-2 (studies by the Paul-Ehrlich-Institute [21, 36]) in the assessment of RAT performance.

A potential confounder in the methodological procedure for specificity assessment is the rather small volume of biological material from the original swab that is actually transferred to the RAT extraction buffer. Here, swab kits containing 1 ml virus transport medium were used, resulting up front in a 1:20 dilution of the input sample. This may have potentially reduced biological components that may trigger a false-positive result. Therefore, the RATs’ specificity of 100% observed in the current study is only informative for the chosen experimental setting to evaluate the analytical sensitivity, but is unlikely to reflect the specificity of the investigated RATs in a point-of-care setting.

The reasons underlying the discrepancy of the RAT performance comparing delta and omicron between respiratory samples, on the one hand, and expanded virus stocks, on the other hand, are currently unclear. As proposed in a cryo-electron tomography study [37], nucleocapsid assembly of SARS-CoV-2 is driven by the intracellular interaction between the nucleocapsid protein and the genomic viral RNA. In a clinical setting, our data suggest that the relative ratio of nucleocapsid protein to SARS-CoV-2 RNA may be higher, on average, in the extracellular space and on the respiratory mucosa of COVID-19 patients infected with delta compared to those infected with omicron.

Several scenarios can be envisioned that may underlie this difference: (1) a variant-specific degree of virus-induced cell death or cytolysis triggered by the host’s immune response may contribute to a VoC-specific level of nucleocapsid protein found on the respiratory mucosa. (2) A higher severity of disease has been associated with increased transcriptional levels of nucleocapsid RNA [38], providing a potential explanation for an enhanced nucleocapsid per viral RNA ratio in COVID-19 as a consequence of a delta infection compared to an omicron infection. (3) Tissue culture-expanded virus stocks are harvested at a time point of considerable lysis of the virus-producing Vero-E6 cells, potentially leveling out VoC-specific differences in the ratio of released nucleocapsid protein relative to viral RNA compared to a more physiological setting. (4) Moreover, one can speculate that SARS-CoV-2-specific antibody responses in COVID-19 patients, either through vaccination or previous infections, could differentially impact on the VoC-specific positivity rate of RATs.

It is widely communicated that individuals with an omicron infection with high viral loads are reliably detected by RATs that are available on the German market, and that these tests’ quality is sufficient to have a positive impact on national pandemic management [39, 40]. These general claims stand in contrast to the following aspects: (1) among the approximately 580 RATs [41] currently available on the German market not even half has been evaluated by either the Paul-Ehrlich-Institute or independent diagnostic laboratories for any SARS-CoV-2 variant, let alone omicron. The Paul-Ehrlich-Institute stated that first results of their omicron validations will be made publically available at the end of February 2022; (2) general claims that reactivity in a RAT reliably identifies the group of “truly” infectious individuals or individuals with super-spreader potential under normal human interaction conditions are not substantiated by published scientific literature. Examples of an apparent super-spreader, long before the emergence of VoCs with enhanced transmissibility, with Ct values of ≥ 27 [42] or cultivation of SARS-CoV-2 from specimen with Ct values ≥ 35 [43, 44] have been reported. Similarly, experimental studies have estimated that around 1000 virus particles may be sufficient for infection of a new host [45, 46], while viral loads required for a positive score in SARS-CoV-2 RATs range more than 1.000-fold higher. (3) The determination of the analytical sensitivity alone is not sufficient to draw conclusions on the clinical performance. Preliminary reports indicate that COVID-19 patients with omicron may shed even less virus than individuals infected with previous VoCs [7]. This could further worsen the clinical performance in light of the lower analytical sensitivity of the majority of RATs examined in this study. Since omicron is considered more contagious than previous VoCs based on epidemiological findings [47, 48], these COVID-19 patients, with or without symptoms, would be assumed to potentially be infectious despite a lower viral load in respiratory swabs. Furthermore, clinical studies are just beginning to assess the level of virus shedding and infectivity in the context of omicron infections and breakthrough infections in vaccinated individuals [7–9, 27].

Modeling studies suggest that the limited sensitivity of RATs to identify infected and infectious individuals may be compensated for by multiple repeated testing. This is based, in part, on claims that a “recalibrated absolute sensitivity” of RATs is around 80% [49]**,** which is, however, not supported by the majority of independent laboratory-based or field studies [26]. On the other hand, especially in the initial phase of symptomatic disease, a time window of a few hours may be sufficient to repeat a rapid antigen testing with low sensitivity in suspected disease to enhance the likelihood of detecting an infection. A recent study suggests that the viral load during this period can rapidly and markedly increase for RATs to score positive [50].

Interestingly, epidemiological studies by the Robert Koch-Institute suggested that 67% of individuals with a negative RAT result believe that they are non-infectious for at least 48 h, potentially affecting their behavior regarding preventive hygiene measures. In part, this perception results from overly optimistic communications on the reliability of this class of tests.

Our current study suggests that the emergence of future VoCs of this pandemic β-coronavirus with an altered mutational pattern in the nucleocapsid protein should result in an immediate reassessment of the performance of RATs. Since the rapidly spreading BA.2 sub-lineage of omicron carries an additional S413R mutation in the nucleocapsid protein, it is difficult to predict the RAT performance for this VoC. The minimum requirements stated by international organizations and the Paul-Ehrlich-Institute for the performance of SARS-CoV-2 rapid antigen tests include an overall diagnostic sensitivity of > 80% and a specificity of > 97% [51, 52]. Independently validated RATs that do not meet these criteria, should be taken off the market immediately.

In light of first international reports [14, 29] and the results from our present study, current communications by the Paul-Ehrlich Institute [51] and the Bundesministerium für Gesundheit [40] on the apparent reliability of RATs for the detection of an omicron infection seem premature. Moreover, critical pre-analytical factors, including the timing of the swab relative to the onset of symptoms, the vaccination status, the swabbing practices and test procedures, in particular when not performed by trained health care professionals in COVID-19 testing centers, may also have a great impact on the “real-world” diagnostic sensitivity of RATs. The latter factors should underlie a constant optimization and a regular and stringent assessment by health authorities.

Vaccination campaigns have achieved a substantial coverage of the adult population in first-world countries, from the perspective of health care systems markedly improving the starting point for the control of future VoCs that may arise from the original evolutionary trait of SARS-CoV-2, from which VoCs alpha, beta, gamma and delta arose. Alternative testing concepts including sample pooling for PCR or isothermal RT-LAMP nucleic acid-based detection of SARS-CoV-2 should be optimized to reduce the potential burden of infections and COVID-19 in the context of large social events with conditions favoring SARS-CoV-2 transmission.

## Supplementary Information

Below is the link to the electronic supplementary material.

Supplementary file1 (PDF 534 KB)
