# Supplementary material for: Impaired detection of omicron by SARS-CoV-2 rapid antigen tests
Source: Med Microbiol Immunol. 2022 Feb 20;211(2-3):105–17. doi: 10.1007/s00430-022-00730-z (PMC8858605; doi:10.1007/s00430-022-00730-z)
Supplement: Supplementary file 1 — Supplementary file1 (PDF 534 KB) [file 430_2022_730_MOESM1_ESM.pdf]

Supplemental Figures

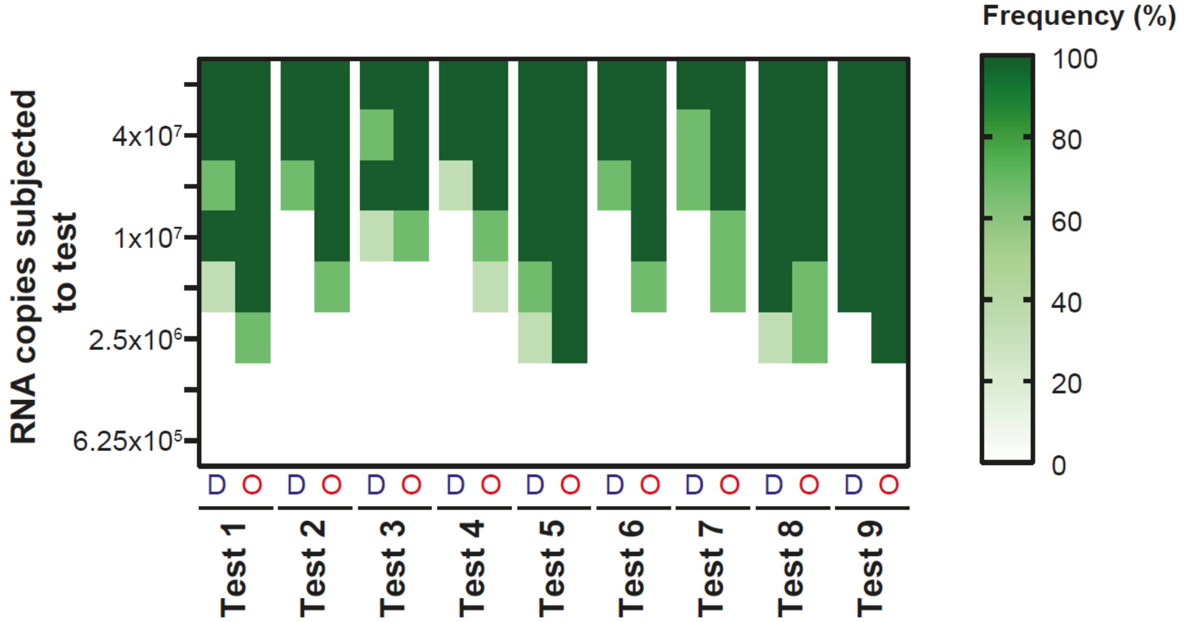

Suppl. Fig. 1 Heat map of analytical sensitivity of nine SARS-CoV-2 RATs for SARS-CoV-2 variants delta and omicron expanded in cell culture. The visual read of triplicate samples were plotted against the calculated viral RNA copies subjected to the test. Dark green: Positive RAT result; Light green: Replicate mixed result; White: Negative RAT result. D: delta; O: omicron.

Supplemental Tables

Suppl. Table 1 Summary of non-synonymous amino acid substitutions in the SARS-CoV-2 nucleocapsid protein with >75% prevalence in publically available VoC sequences compared to the original Wuhan-hu-1 sequence [13].

Amino acid position	D3	P13	DEL31/33	D63	P80	R203	G204	T205	G215	A220	S235	D377	S413
Alpha (B.1.1.7)	L					K	R				F		
Beta (B.1.351)								I					
Gamma (P.1)					R	K	R						
Delta (B.1.617.2)				G		M			C			Y	
Omicron (BA.1)		L	del*			K	R						
Omicron (BA.2)		L	del*			K	R						R

*deletion

Suppl. Table 2 Detailed information of the nine SARS-CoV-2 RATs examined in this study.

**Testnumber**	**1**	**2**	**3**	**4**	**5**	**6**	**7**	**8**	**9**
**Manufacturer**	FUJIFILM Corporation	Beijing Hotgen Biotech Co., Ltd.	NanoRepro AG	Healgen Scientific LLC	Hangzhou Laihe Biotech Co., Ltd.	Biosynex Swiss SA	Xiamen Boson Biotech Co., Ltd.	MP Biomedicals Germany GmbH	Xiamen Boson Biotech Co., Ltd.
**Distributor**		TREKSTOR GmbH	Viromed GmbH	Siemens Healthineers AG	Lissner Qi GmbH		Technomed GmbH		Medice Arzneimittel Pütter GmnB & Co. KG
**Autorised representative**	FUJIFILM Europe GmbH	MedNet GmbH		Shanhai International Holding Corp. GmbH	SUNGO Europe B.V.		Lotus NL B.V.		
**Test name**	FUJIFILM COVID-19 Ag Test	Novel Coronavirus 2019-nCoV Antigen Test (Colloidal gold)	NanoRepro SARS-CoV-2 Antigen Schnelltest (Viromed)	CLINITEST Rapid COVID-19 Antigen Test	Lyher Novel Coronavirus (COVID-19) Antigen Test Kit (Colloidal Gold)	COVID-19 Ag BSS self-test	Rapid SARS-CoV-2 Antigen Test Card	Rapid SARS-CoV-2 Antigen Test Card	Medicovid-AG SARS-CoV-2 Antigen Rapid Test Card - nasal
**Ref. No.**	16696722	HGCG134S0101	B66200	GCCOV-502a	303036	859270	1N40C5	07AG6001BS	1N40C5-4
**Used antibodies**	Monoclonal	Monoclonal	Monoclonal	Monoclonal	Monoclonal	Monoclonal	Monoclonal	Monoclonal	Monoclonal
**Detected antigen**	Nucleocapsid	Nucleocapsid	Nucleocapsid	Nucleocapsid	Nucleocapsid	Nucleocapsid	Nucleocapsid	Nucleocapsid	Nucleocapsid

**Recommended material**	Nasopharyngeal swab	Nasal swab	Nasal swab	Nasopharyngeal swab	Nasal swab	Nasal swab	Nasopharyngeal swab	Nasal swab	Nasal swab
**PEI evaluated (31.01.22)**	yes (as professional test)	yes (as self-test)	yes[$]	yes (as self-test)	yes (as self-test)	yes* (as professional test)	yes* (as self-test)	yes (as professional test)	no
**Intended by the manufacturer for self-testing ("self-tests")**	no - intended for professional use ("rapid tests")	yes	yes	yes	yes	yes	yes	yes	yes
Manufacturer Sensitivity	94%	95,37%	97,33%	relative: 96.72%	95%	97,20%	93,75	96,19%	96,19%
Manufacturer Specificity	100%	99,13%	99,33%	relative: 99.22%	99%	100%	98,04	99,20%	99,20%
Manufacturer Limit of detection [$TCID_{50}$]	1.1 x 10^2 $TCID_{50}$/ml	-	30 TCID50/ml	1.15 x10^2 $TCID_{50}$/ml	0.5ng/ml	-	1.3 x10^2 $TCID_{50}$/ml<br>1ng/ml	-	1.3 x10^2 $TCID_{50}$/ml<br>1ng/ml

*listed in the "SARS-CoV-2 antigen rapid diagnostic tests passing the sensitivity criteria" of the Paul-Ehrlich-Institute (date of access: 31.1.22) with different reference Nos. [$] [30]

Suppl. Table 3 Determination of assay specificities for nine SARS-CoV-2 RATs using SARS-CoV-2 PCR-negative respiratory swabs

Assay	Specificity (%)	95% CI (%)			True negative / total		
Test 1	100	96.71	-	100	113	/	113
Test 2	100	96.74	-	100	114	/	114
Test 3	100	96.74	-	100	114	/	114
Test 4	100	96.65	-	100	111	/	111
Test 5	100	96.77	-	100	115	/	115
Test 6	100	96.34	-	100	101	/	101
Test 7	100	96.74	-	100	114	/	114
Test 8	100	96.74	-	100	114	/	114
Test 9	100	96.53	-	100	107	/	107

Binomial confidence intervals were computed using the Wilson score interval
